
JOURNAL INFORMATION
==============================
NLM Title Abbreviation: ScientificWorldJournal
Journal ID: ScientificWorldJournal
NLM Journal ID: 101131163
Journal ID: TSWJ

The Scientific World Journal

ISSN: 2356-6140
EISSN: 1537-744X
Publisher: Wiley

ARTICLE INFORMATION
==============================
PMCID: PMC6084205
PMID: 30147559
DOI: 10.1100/tsw.2001.206
Article ID: 845097
Article version: 1
Subjects: Short Report

The Relevance of Oncogene-Induced Apoptosis in Cancer

Evan Gerard
UCSF Cancer Center, 2340 Sutter Street, San Francisco, California 94143-0128, USA
Publication date: 2001
Electronic publication date: 2001 Dec 19
Volume: 1
First page: 89
Last page: 89
Copyright: Copyright © 2001 Gerard Evan.
Copyright year: 2001
License: This is an open access article distributed under the Creative Commons Attribution License, which permits unrestricted use, distribution, and reproduction in any medium, provided the original work is properly cited.
License URL: https://creativecommons.org/licenses/by/3.0/

==============================
Miami *Nature Biotechnology* Short Reports
*TheScientificWorld* (2001) 1 (S3), 89SR
ISSN 1532-2246; DOI 10.1100/tsw.2001.206

### THE RELEVANCE OF ONCOGENE-INDUCED APOPTOSIS IN CANCER

Gerard Evan

UCSF Cancer Center, 2340 Sutter Street, San Francisco, California 94143-0128, USA

Cancers arise through the progressive accumulation of mutations that compromise control of cell proliferation, differentiation, cell adhesion, apoptosis and mortality. Deregulation of the c-*myc* proto-oncogene is a ubiquitous neoplastic mutation that disrupts cell growth control and renders cells independent of mitogens for cell cycle progression. However, activation of c-*myc* also sensitises cells to apoptosis. We believe that this innately contradictory action of c-*myc* acts as a restraint to the propagation of neoplastic cells within the soma, but this has never been directly demonstrated *in vivo*.

To investigate the relevance of apoptosis in the suppression of c-*myc*-induced carcinogenesis, we have constructed mice harbouring a switchable c-Myc protein in specific tissues - specifically, pancreatic islets cells and suprabasal keratinocytes. This has allowed us to examine *in vivo* both the immediate and the delayed consequences of acute activation of the c-*myc* oncogene, in different somatic settings. In pancreatic β cells, c-Myc activation induces essentially 100% entry of cells into cell cycle. However, this is accompanied by massive apoptosis that overwhelms proliferation and leads to rapid islet involution and concomitant acute diabetes. The clear implication is that in β cells neoplasia could not arise without early suppression of apoptosis. To formally demonstrate the oncogenic synergy between c-*myc* and suppression of apoptosis in β cells, we have co-expressed the anti-apoptotic Bcl-$x_L$ protein in β cells together with switchable c-Myc. Upon activation of c-Myc, we observe dramatic, progressive and inexorable expansion of β cells in all islets, leading to angiogenic and invasive adenomas that progress to metastatic carcinoma. Thus, in pancreatic β cells, deregulated proliferation and suppressed apoptosis appears to be all that is necessary to generate a full blown cancer.

By contrast, activation of c-Myc in suprabasal skin triggers cell proliferation and blockade of keratinocyte differentiation, but no attendant and “compensatory” apoptosis is evident. Keratinocyte hyperplasia is progressive, and within three weeks leads to formation of a complex parakeratotic papilloma that exhibits blocked keratinocyte differentiation, focal dysplasia and neo-angiogenesis. The surprising absence of apoptosis appears to be due to an abundance of anti-apoptotic survival factors in intact skin that act to suppress the otherwise toxic action of oncogenic c-Myc. The strong implication from this is that, unlike β cells, suppression of apoptosis through mutation is not required for the establishment and progression of epidermal skin tumours.

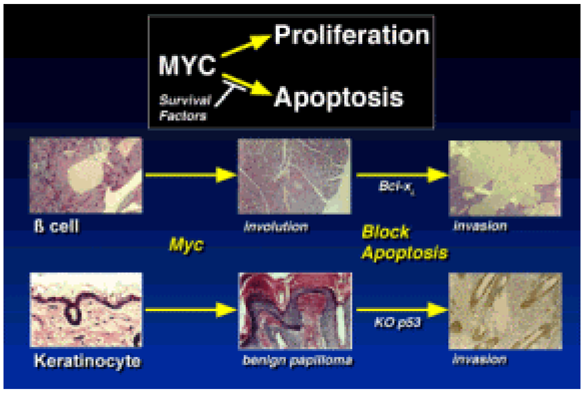

Because the switchable c-Myc system we use is reversible, we are also able to examine the consequences of c-Myc ablation on our tumor models: in effect, we can test genetically what might be the effect on tumors of pharmacological interference with c-Myc function. In skin, inactivation of c-Myc leads to rapid regression of c-Myc-induced papillomas and the consequent reappearance of skin that is indistinguishable from normal. We are currently investigating the mechanisms underlying this rapid regression in order to establish optimum molecular targets whose modulation might trigger analogous regression in human skin cancers.

One intriguing feature of our c-Myc-induced papillomas is that they remain completely non-invasive. However, in the absence of p53 they rapidly invade the underlying dermis and mesenchyme. As p53 is required for efficient c-Myc-induced apoptosis in keratinocytes, the primary role for p53 loss appears to be to allow survival of invading keratinocytes when migrating into an “inappropriate” trophic environment. This, again, underscores the fundamental importance that suppression of apoptosis plays in the development and progression of cancer. Moreover, our two transgenic models exemplify how different “rules” govern tumorigenesis and progression in tissues reflecting differing architectures and functions and may shed light on why neoplasms in differing locations adopt preferred evolutionary routes to malignancy.
